# Sonographie als Gegenstand virtuellen Wissenstransfers zum praktischen Kompetenzerwerb

**DOI:** 10.1007/s00106-024-01476-1

**Published:** 2024-04-08

**Authors:** F. Everad, L. Seifert, N. Mansour, B. Hofauer, A. Knopf, C. Offergeld

**Affiliations:** 1 Klinik für Hals- Nasen- und Ohrenheilkunde, Universitätsklinikum Freiburg, Killianstraße 5, 79106 Freiburg, Deutschland; 2 Universitätsklinik für Hals‑, Nasen- und Ohrenheilkunde Innsbruck, Innsbruck, Österreich

**Keywords:** Digitale Technologie, Lehre, Online-Lernen, Medizinstudierende, Simulationstraining, Digital technology, Teaching, Online learning, Medical students, Simulation training

## Abstract

**Zielsetzung:**

Während der COVID-19-Pandemie bestand eine besondere Herausforderung bei der Umstellung auf den digitalen Unterricht darin, praktische Fertigkeiten wie die Sonographie der Kopf- und Halsweichteile online zu vermitteln. Ziel dieser Studie war es, den an der Universitäts-HNO-Klinik Freiburg etablierten digitalen Sonographie-Kurs für Studierende der Humanmedizin zu validieren.

**Methoden:**

Teilnehmende waren 178 Studierende der Humanmedizin. Die Studiengruppe simulierte die Sonographie-Untersuchung zu Hause mit einer Schallkopfattrappe anhand der Peyton-Methode unter Anleitung eines Tutors per Videoseminar. In einer anschließenden Lernerfolgskontrolle wurden die Ergebnisse der Studierenden des Online-Kurses mit der Kontrollgruppe verglichen, welche die Sonographie im Präsenzunterricht lernte.

**Ergebnisse:**

Die Studierenden des Online-Kurses konnten vergleichbare Ergebnisse zur Präsenzgruppe erzielen.

**Schlussfolgerung:**

Die Studie zeigt, dass praktische Fertigkeiten, die eine umfangreiche Ausrüstung wie ein Sonographiegerät erfordern, bis zu einem gewissen Grad digital oder zumindest in einer hybriden Form vermittelt werden können.

**Zusatzmaterial online:**

Die Online-Version dieses Beitrags (10.1007/s00106-024-01476-1) enthält den Dokumentationsbogen der Lernerfolgs-Checkliste.

## Abstract (en)

**Objective:**

During the COVID-19 pandemic, a particular challenge in the transition to digital teaching was to teach practical skills such as sonography of the head and neck online. The aim of this study was to validate the digital sonography course for medical students established at the Freiburg University Hospital ENT Department.

**Methods:**

Participants were 178 students of human medicine. The study group simulated the sonography examination at home with a dummy transducer using the Peyton method under the guidance of a tutor via video seminar. In a subsequent learning success check, the results of the students in the online course were compared with those of the control group, who learned sonography in the classroom.

**Results:**

Students of the online course achieved comparable results to the classroom group.

**Conclusion:**

This study shows that practical skills which require extensive equipment such as a sonography machine can be taught to a certain extent digitally or at least in a hybrid form.

Die Vermittlung praktischer Fertigkeiten war eine der größten Herausforderungen der Digitalisierung des HNO-Curriculums während der COVID-19-Pandemie. Gleichwohl zeigten Online-Untersuchungskurse mit der Bereitstellung von Videos, digitalen Materialien und Skripten erste positive Ergebnisse. Die Kopf-Hals-Sonographie stellte jedoch aufgrund mangelnder technischer Infrastruktur, wie mobilen Sonographiegeräten, eine besondere Herausforderung dar. Mit einem digitalen Sonographiekurs soll diese gemeistert werden.

## Neustrukturierung der Lehre

Durch die COVID-19-Pandemie musste die universitäre Lehre vollständig neu strukturiert werden. Die Umsetzung von Vorlesungen und Seminaren in digitale Formate gelang den meisten nationalen HNO-Kliniken bereits im ersten COVID-Semester [1]. Die Lehre von theoretischen Inhalten in diversen Lehrformaten wurde von den Studierenden teilweise sogar als Zugewinn zur klassischen Präsenzvorlesung gesehen [2]. Indes ließ sich die Vermittlung praktischer Fertigkeiten nicht unverzüglich in ein digitales Format umsetzen und wurde als Limitation der digitalen Lehre gesehen [2]. Gleichwohl setzten Krauss et al. erfolgreich ein Videokonferenzsystem sowie Einmalinstrumente zur Durchführung des „HNO-Spiegelkurses“ ein [3]. Die digitale Vermittlung der Kopf-Hals-Sonographie war bislang nicht Gegenstand von Forschungsstudien.

Die immer relevanter werdende Ultraschalldiagnostik hat bereits Einzug in den Nationalen Kompetenzbasierten Lernzielkatalog Medizin (NKLM) gefunden [4]. Der NKLM definiert ein Lernziel als die Fähigkeit „unter Anleitung selbstständig eine orientierende Sonographie […] des Halses […] durchzuführen.“ Die Ausweitung der sonographischen Ausbildung von Medizinstudierenden ist Diskussionsthema vor dem Hintergrund einer steigenden Relevanz im ärztlichen Alltag [5, 6]. Dessen ungeachtet konnte die Kopf-Hals-Sonographie während der COVID-19-Pandemie aufgrund fehlender technischer Infrastruktur (z. B. mobile Sonographiegeräte) vielerorts nicht digital gelehrt werden. Aus diesem Grund etablierte die Klinik für Hals‑, Nasen- und Ohrenheilkunde am Universitätsklinikum Freiburg 2020 einen digitalen Sonographiekurs nach der Methode des Inverted Classroom [7]. Ziel dieser Studie war es, dessen Umsetzbarkeit und Effektivität im Vergleich zum klassischen Präsenzunterricht am Sonographiegerät zu untersuchen.

## Methoden

### Studiendesign

Es handelt sich um eine monozentrische, prospektive, kontrollierte Studie. Teilnehmende waren Studierende der Humanmedizin, die im Sommersemester 2021 das HNO-Blockpraktikum absolvierten. Eine Randomisierung war aufgrund der zum Studienzeitpunkt geltenden universitätsinternen COVID-Vorschriften nicht möglich, da die Präsenzgruppe mindestens einen zusätzlichen Personenkontakt (mit dem Tutor) hatte und somit die Teilnehmer bei einer Randomisierung „gezwungen“ wären, dieses erhöhte gesundheitliche Risiko einzugehen. Aus diesem Grund ließen die Autoren die Teilnehmer zwischen Studiengruppe (Teilnahme vorwiegend online) und Kontrollgruppe (Teilnahme vorwiegend in Präsenz) wählen. Ein positives Votum der lokalen Ethikkommission (EK 21-1341) lag zum Studienbeginn vor.

### Lehrkonzepte

In dieser Studie wurden 2 Lehrkonzepte angewendet; das Konzept des Inverted Classroom [8] zur Strukturierung des Unterrichts und die 4‑Schritt-Methode nach Peyton [9] zum Erlernen der Kopf-Hals-Sonographie.

#### Inverted Classroom

Im Inverted Classroom erfolgt die Vermittlung des theoretischen Inputs – im Gegensatz zum klassischen Unterricht – zu Hause im Selbststudium, z. B. mittels Lehrvideos. Im anschließenden Präsenzunterricht können die Studierenden ihr theoretisches Wissen dann direkt praktisch anwenden.

#### Peyton-Methode

Die Fertigkeit selbst wird mithilfe der Peyton-Methode in 4 Schritten erlernt:

1. Demonstration,
2. Dekonstruktion,
3. Verständnis und
4. Durchführung.

Hierdurch können in Verbindung mit der Methode des Inverted Classroom praktische Fertigkeiten digital vermittelt werden. Die Schritte 1–3 nach Peyton lassen sich im Sinne des Inverted Classroom im Heimstudium durchführen, und lediglich Schritt 4 (das Durchführen) erfordert eine tatsächliche Anwesenheit zur Überprüfung des Lernerfolgs.

### Studienablauf

**Figure 1:**
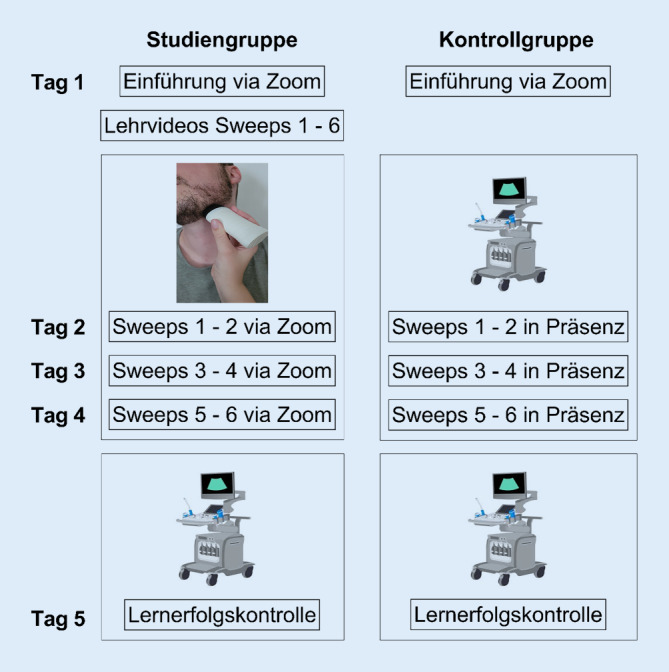

**Figure 2:**
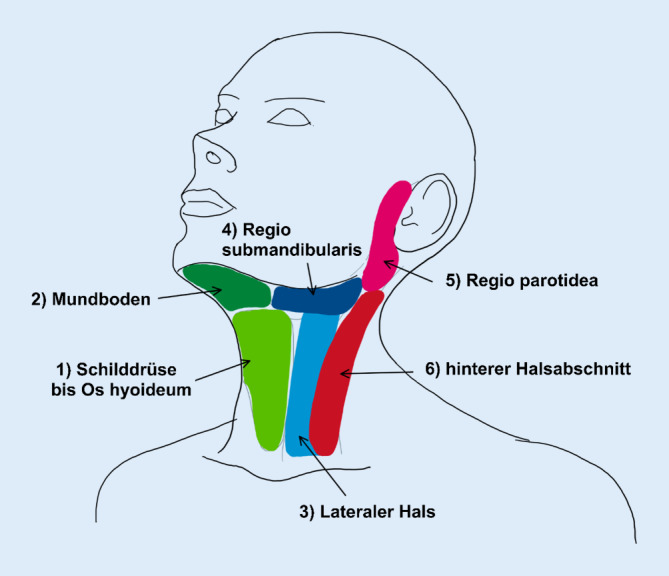

**Figure 3:**
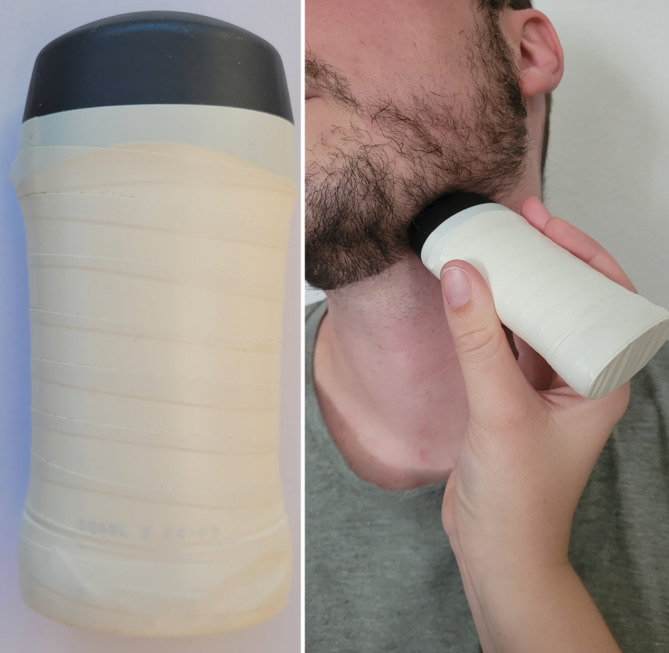

Die Studie erfolgte im Rahmen des HNO-Blockpraktikums und bestand aus 5 Studientagen (Abb. 1). Die teilnehmenden Studierenden wurden jeweils in eine Studiengruppe (SG) und eine Kontrollgruppe (KG) eingeteilt. Für beide Gruppen erfolgte an Tag 1 eine Einführung in die Kopf-Hals-Sonographie in einem 45-minütigen Online-Seminar mit dem Fokus auf den Grundlagen der Anatomie, Sonographie und deren Befundung im Kopf-Hals-Bereich. Der Kopf-Hals-Bereich wurde dabei in 6 sonographierelevante Regionen („Sweeps“) aufgeteilt (Abb. 2). Die Studierenden der SG erhielten im Anschluss einen Link zu Lehrvideos, um im Selbststudium die Sweeps zu erarbeiten. An Tag 2–4 führten die Studierenden der SG die Sweeps unter Anleitung eines Tutors über ein kommerzielles Videokonferenzsystem (Zoom, Fa. Zoom Video Communications Inc, San Jose, CA, USA) durch. Hierfür nutzen sie zuvor ausgegebene Schallkopfattrappen (Abb. 3) und Ultraschallgel. Während die Studierenden einen Halsabschnitt mit der Schallkopfattrappe „sonographierten“, wurde ihnen das entsprechende Sonographiebild im B‑Bild-Modus via Zoom gezeigt. An jedem Studientag wurden 2 neue Regionen erlernt sowie die bereits erlernten wiederholt.

Im Vergleich dazu erlernte die KG die Sweeps an den Tagen 2–4 in 15-minütigen Unterrichtseinheiten am Sonographiegerät. Beide Gruppen führten an Tag 5 die vollständige sonographische Untersuchung am Sonographiegerät im Rahmen einer Lernerfolgskontrolle durch.

### Lernerfolgskontrolle

Die Lernerfolgskontrolle erfolgte mithilfe einer Checkliste (*Online-Zusatzmaterial*), auf der alle zu erreichenden Ziele definiert und mit einer Punktzahl versehen waren (z. B. Darstellung der A. carotis communis im Horizontalschnitt, 2 Punkte). Geprüft wurden die sonographischen Grundlagen (z. B. eine echoreiche Struktur im Kopf-Hals-Bereich zeigen) sowie die Durchführung aller 6 Sweeps. Zudem wurde die Einhaltung der Reihenfolge und Vollständigkeit der Gesamtuntersuchung bewertet. Besonders anspruchsvolle Aufgaben (z. B. die Darstellung des Os hyoideum im Horizontalschnitt) wurden mit Bonuspunkten bemessen. Insgesamt konnten 61 reguläre Punkte und 11 Bonuspunkte erreicht werden.

### Statistische Auswertung

Die Daten wurden mit der Statistiksoftware Graphpad Prism (Version 10, © 2023 GraphPad Software, https://www.graphpad.com/) analysiert. Nach Testung auf Normalverteilung wurden signifikante Gruppenunterschiede anhand von t‑Tests (parametrische Daten) und Wilcoxon-Tests (nichtparametrische Daten) ermittelt. Ein Signifikanzniveau wurde ab einem *p*-Wert von < 0,05 erreicht. Bei signifikanten Unterschieden wurde zur Veranschaulichung einer praktischen Relevanz die Effektstärke Cohen‑d errechnet (d < 0,5: kleiner Effekt, d = 0,5–0,8: mittelgradiger Effekt, d > 0,8: großer Effekt). Die deskriptive Datenanalyse umfasste die Ermittlung des Mittelwerts und der Standardabweichung.

## Ergebnisse

### Studiendesign

In die Studie wurden 178 Studierende eingeschlossen. Aufgrund einer unvollständigen Kursteilnahme wurden 3 Datensätze exkludiert, wodurch sich eine Gesamtzahl von 175 Datensätzen ergab. Die Studiengruppe (SG) bestand aus 83 Teilnehmern, die Kontrollgruppe (KG) aus 92 Teilnehmern.

### Vergleich Online- vs. Präsenzgruppe

#### Durchführung der sonographischen Grundlagen

Die Studierenden der SG erreichten im Grundlagenteil durchschnittlich 5,9 ± 0,431 Punkte der möglichen 6 Punkte, die KG erreichte im Durchschnitt 5,7 ± 0,835 Punkte. Damit erreichte die Online-Gruppe im Grundlagenteil ein statistisch signifikant besseres Ergebnis (*p* = 0,019; Cohen-d = 0,308). Die maximalen Bonuspunkte im Bereich Grundlagen konnten in beiden Gruppen von allen Studierenden erreicht werden. In der Einhaltung der Reihenfolge und Vollständigkeit der Gesamtuntersuchung zeigten sich ebenfalls keine signifikanten Unterschiede.

#### Durchführung der 6 Sweeps

**Figure 4:**
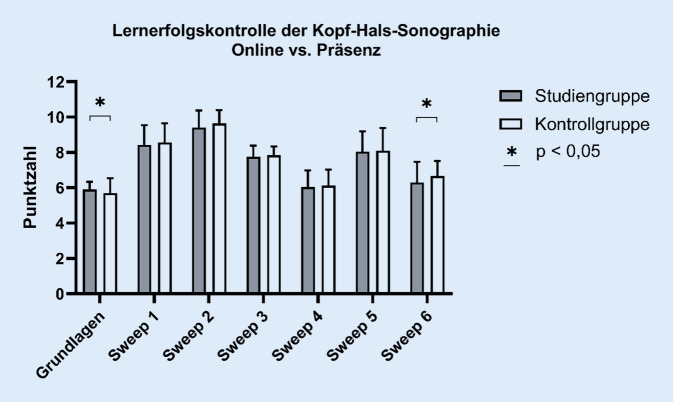

In den 6 Sonographieabschnitten zeigten sich nur geringe Leistungsunterschiede (Abb. 4). Nur im 6. Sweep (hinterer Halsabschnitt) erreichte die KG ein statistisch signifikant besseres Ergebnis (*p* = 0,009, Cohen-d = 0,369). In den weiteren Sweeps konnte bei den regulären Punkten kein weiterer Unterschied festgestellt werden.

Bei der Verteilung der Bonuspunkte erzielte die Präsenzgruppe in 2 von 6 Schnitten ein besseres Ergebnis als die Studiengruppe. Im direkten Vergleich wurde im 2. Sweep (Mundboden) ein *p*-Wert von 0,013 (Cohen-d = 0,336) und im 3. Sweep (lateraler Hals) ein *p*-Wert von < 0,001 (Cohen-d = 0,464) festgestellt.

#### Gesamtpunktzahl und Gesamtbonuspunkte

**Figure 5:**
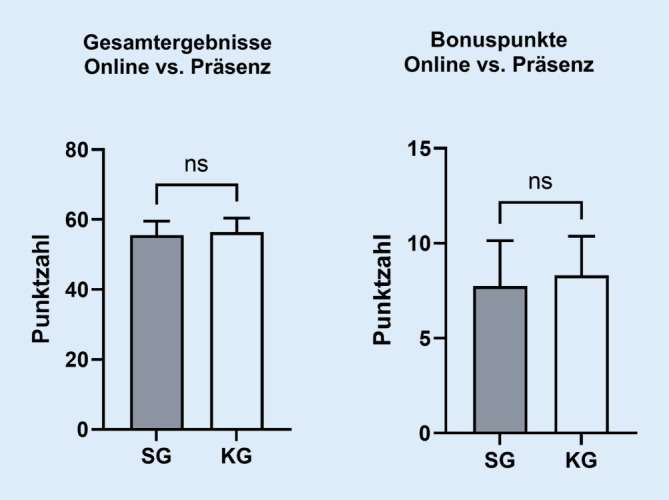

Betrachtet man die Gesamtergebnisse, so lässt sich keine statistisch signifikante Überlegenheit zwischen der Online- und der Präsenzgruppe feststellen (*p* = 0,078; Abb. 5). Die SG erreichte im Durchschnitt 55,52 ± 4,127, die KG 56,40 ± 4,074 der maximalen 61 Punkte. Von den Bonuspunkten erzielte die SG im Mittel 7,8 ± 2,378 Punkte, während die KG durchschnittlich 8,30 ± 2,069 Punkte erreichte. Im Gruppenvergleich zeigte sich auch hier keine statistische Signifikanz (*p* = 0,066). Zusammenfassend erreichte die Online-Gruppe durchschnittlich 91 %, die Präsenzgruppe 92 % der Gesamtpunktzahl. Damit ließ sich keine statistisch signifikante Überlegenheit einer Gruppe feststellen.

## Diskussion

Die Deutsche Gesellschaft für Ultraschall in der Medizin (DEGUM) geht davon aus, dass primär theoretische Inhalte zum Ultraschall erfolgreich digital vermittelt werden können. Die Möglichkeit, auch praktische Inhalte in gleicher Weise zu lehren, wird kritisch gesehen, wenngleich die Aufforderung zur Präsentation kreativer Konzepte vonseiten der DEGUM als Motivationsanreiz angesehen werden kann.

Die vorliegenden Ergebnisse belegen, dass mithilfe eines strukturierten Online-Kurses eine effektive Vermittlung der praktischen Kopf-Hals-Sonographie gelingen kann. Die sowohl von der Online- als auch der Präsenzgruppe erreichten durchschnittlichen Testscores (SG 91 % vs. KG 92 %) zeigen, dass beide Lehrformate vergleichbar erfolgreich zu sein scheinen. Auch im Vergleich mit bereits publizierten Ergebnissen einer lehrmethodisch differenten, aber thematisch vergleichbaren Erhebung ist die durchschnittliche Gesamtpunktzahl in einem vergleichbaren Maße als hoch einzustufen [3].

### Grundlagenteil

Fokussiert man nun auf einzelne Teilbereiche der Sonographie, so lassen sich spezifische Charakteristika zwischen Online- und Präsenzgruppe ableiten. Im Grundlagenteil konnte die SG durchschnittlich besser punkten als die KG. Dies beinhaltet u. a. die Orientierung am Gerät und Fragen zur Echogenität. Eine mögliche Erklärung könnte in der sehr dezidierten Vermittlung der Orientierung am Gerät bzw. Unterweisung in der SG liegen, welche sich besonders durch die intensive Kommunikation ergab. Somit gingen die Beschreibungen und Anleitungen in der SG in eine verbal implementierte Handlungsroutine über.

### Echtzeitdarstellung

Die mangelnde Echtzeitdarstellung ohne ausreichendes visuell-taktiles Feedback ist eine Limitation eines digitalen Ultraschallkurses [10]. Dies spiegelte sich in der schwächeren Ultraschalldurchführung des 6. Sweeps „lateraler Hals“ der Studiengruppe wider. Die Darstellung einer pulsierenden A. carotis communis lässt sich offensichtlich eingängiger am bewegten Bild realisieren als durch eine „Trockenübung“ unter verbaler Anleitung im virtuellen Raum. Hier können die Hypothesen von Polk et al. zum Tragen kommen, welche postulieren, dass eine Redundanz von praktischen Lerninhalten nach einer definierten Zahl von Wiederholungen zum effektiven Lernerfolg führen kann [11]. Ähnliche Ergebnisse zeigten sich in der Identifikation von „schwierigen“ beweglichen Strukturen, welche im sonographischen Echtzeitbild einen hohen Wiedererkennungswert aufweisen (z. B. Zunge, M. omohyoideus). Hier offenbaren sich Vermittlungsprobleme des digitalen Kurses. Dass „schwierige“ praktische Fertigkeiten digital wesentlich häufiger durchgeführt werden müssen, um einen vergleichbaren Kompetenzstatus zu erreichen wie in der Präsenzlehre, zeigten bereits Krauss et al. [3].

### Didaktische Schwierigkeiten

In der Durchführung der Online-Übungseinheiten zeigten sich im Verlauf einige didaktische Schwierigkeiten. Durch die Barriere des virtuellen Raums war ein hochstrukturierter und standardisierter Ablauf der Unterrichtseinheit sowie eine aktive Integration aller teilnehmenden Studierenden in den Lernprozess essenziell. Es benötigte die visuelle Darstellung anhand standardisierter Präsentationen sowie die strikte Einteilung in Kleingruppen, um eine effektive Lernatmosphäre zu schaffen. Durch aktive Einbindung der Studierenden der SG, u. a. im offenen Diskussionsforum, konnte eine aktive Lernsituation erzeugt werden mit dem Ziel, den Lernerfolg zu maximieren [7]. Die Kernpunkte dieses Lehrmodells bestanden demnach aus theoretischer Wissensvermittlung, Ausführung praktischer Tätigkeiten nach Vorgabe der Peyton-Methode und einer ständigen Repetition von Lehrinhalten. Dieser medizindidaktisch wichtige Parameter ist sowohl für die Präsenzlehre [11] als auch für das Online-Pendant dieses Lehrformats von übergeordneter Bedeutung [3] und wurde durch die hier gemachten Erfahrungen bestätigt. Es zeigte sich jedoch ein hoher Ressourcenaufwand, die Betreuung der Kleingruppen von max. 4 Studierenden erfolgte durch studentische Tutor:innen unter ständiger Supervision eines DEGUM-III-Status-Inhabers. Diese Integration von Studierenden in die Lehre hat sich dabei als effektives Mittel erwiesen [12, 13], so auch in der vorliegenden Studie. Diese durchlaufen eine intensive fachspezifische und medizindidaktische Ausbildung. Für die Durchführung eines digitalen Kurses gibt es bisher jedoch keine konkreten Angebote für studentische Tutor:innen. Insofern stellt diese Studie nicht nur bezüglich der Kopf-Hals-Sonographie ein Pilotprojekt dar, sondern auch im Hinblick auf die digitale Transformation der (Peer‑)Lehre [14–16].

### Randomisierung

Methodenkritisch muss festgehalten werden, dass die zum Zeitpunkt der Studie herrschende Pandemielage keine Randomisierung zuließ. Zur Einhaltung der Hygienevorschriften und Kontaktreduktion erfolgte die Einteilung in die Gruppen sowie Übungspaare nach Studierendenwunsch. Dies kann einen Selektionsbias zur Folge haben, da Studierende, die den Präsenzkurs wählen, eventuell eine höhere Lernbereitschaft aufweisen.

### Digitale Ultraschallsimulatoren

Die vorliegenden Auswertungen beziehen sich auf die Ausbildung im absoluten Basisniveau, d. h. sonographische Novizen. Es wurde gezeigt, dass durch einen strukturierten Online-Kurs unter Anwendung von „Trockenübungen“ Basisgrundlagen der Kopf-Hals-Sonographie nicht nur theoretisch, sondern auch praktisch effektiv vermittelt werden können. Ob sich die Aussagekraft der vorliegenden Daten auch auf das Erlernen anderer Regionen respektive Ultraschalluntersuchungen in Online-Kursen ausweiten lässt, ist unter der Berücksichtigung des allgemeinen Settings aktuell nicht eindeutig zu beantworten. Konkrete Daten zur Komplexität der verschiedenen Ultraschalluntersuchungen und der unterschiedlichen Schweregrade dieser Durchführung sind den Autoren nicht bekannt. Allerdings zeigte ein Kurzbeitrag über den angepassten Ultraschallkurs, welcher u. a. die Abdomen- und FAST-Sonographie beinhaltet, des „sonoBYstudents-Programms“ an der medizinischen Fakultät der Universität des Saarlandes bereits die positiven Erfahrungen mit einem an die Pandemie angepassten Kurskonzept [17]. Ein rein digitaler Sonographiekurs für Studierende ist in der Literatur dabei noch nicht beschrieben. Die immer einfachere Verfügbarkeit von digitalen Ultraschallsimulatoren stellt dabei eine nicht zu vernachlässigende Möglichkeit für die Ultraschallausbildung von Studierenden dar. Diese wurden in ersten Studien bereits positiv bewertet [18, 19] und sollten weiter auf ihren Nutzen für die Lehre geprüft werden. Um in der studentischen Ausbildung mit der Digitalisierung Schritt zu halten, sind weitere Untersuchungen zur digitalen Lehre praktischer Fertigkeiten erforderlich.

## Fazit für die Praxis

- Die Grundlagen der Sonographie und die Durchführung einer Kopf-Hals-Sonographie (Theorie und Praxis) können digital effektiv vermittelt werden.
- Eine Standardisierung der Lehreinheiten, die Verwendung von anerkannten Lehrmethoden und die häufige Wiederholung sind unverzichtbar.
- Die Durchführung des Online-Sonographiekurses benötigt hohe personelle Ressourcen sowie eine hohe didaktische und fachliche Ausbildung des Tutor:innen-Personals.
- Die Vermittlung von Strukturen, die durch ihre Bewegung im dynamischen Sonographiebild charakterisiert sind, scheint eine Limitation des Online-Kurses darzustellen.
- Der Bedarf an strukturierter Ultraschallausbildung ist hoch, ein DEGUM-zertifizierter ausschließlich online durchgeführter Ultraschallkurs für Studierende existiert aktuell jedoch nicht.

### Supplementary Information




