# Supplementary material for: Virtual training of practical competences in sonography
Source: HNO. 2024 Apr 8;72(5):350–6. [Article in German] doi: 10.1007/s00106-024-01476-1 (PMC11045615; doi:10.1007/s00106-024-01476-1)
Supplement: Supplementary file 1 [file 106_2024_1476_MOESM1_ESM.pdf]

Online-Zusatzmaterial: Dokumentationsbogen der Lernerfolgs-Checkliste

**Kopf-Hals-Sonographie Sommersemester 2021**	**Datum:**
**Vorerfahrung Sonographie: Ja◯ Nein◯**	
**Klinisches Semester:**	**Gruppe: A ◯ B ◯**

Einführung

**Ultraschall allgemein**	falsch	Abzüge
Echogenität:		
• Alle aufzählen	◯	-2
• Beispiel für **EINE** der Echogenitäten abfragen, also entweder: a) Echoreich (z.B. Drüse) b) Echoarm (z.B. Muskel) c) Echoleer (z.B. Gefäß)	◯	-2
Orientierung am Ultraschallgerät/ -bild: (Fingertest horizontal und vertikal)	◯	-2
**Bonuspunkt:** US-Gel aufgetragen	◯	**+1**
**Abzug gesamt:**		**/ 6**
**Bonuspunkte:**		**/ 1**

1) Schnitt

**Schilddrüse bis Os hyoideum**	falsch	Abzüge
Schilddrüse:		
• Schallkopfhaltung horizontal	◯	-2
• Strukturen erkennen:		
▪ Schilddr: Isth/SDL bds	◯	-2
▪ A. carotis communis	◯	-1
▪ V. jugularis interna	◯	-1
▪ M. sternocleidomast.	◯	-2
▪ Trachea	◯	-1
▪ **Bonus**: Ösophagus	◯	**+1**
▪ **Bonus:** Os hyoideum	◯	**+2**
▪ **Bonus:** Sonopalpation	◯	**+1**
**Abzug gesamt:**		**/ 9**
**Bonuspunkte:**		**/ 4**

2) Schnitt

**Mundboden**	falsch	Abzug
• Schallkopfhaltung horizontal	◯	-2
• Schallkopfhaltung vertikal	◯	-2
• Darstellung horizontal:		
▪ M. mylohyoideus	◯	-1
▪ M. geniohyoideus	◯	-1
▪ M. genioglossus	◯	-1
▪ M. digastricus (beidseits)	◯	-2
▪ **Bonus:** Mickey Mouse	◯	**+1**
▪ **Bonus:** Zungenoberfl	◯	**+1**
• Darstellung vertikal:		
▪ Mandibula	◯	-1
▪ **Bonuspunkt:** Os hyoid	◯	**+1**
**Abzug gesamt:**		**/ 10**
**Bonuspunkte:**		**/ 3**

3) Schnitt

**Lateraler Hals**	falsch	Abzüge
• Schallkopfhaltung horizontal	◯	-2
• Darstellung:		
▪ A. carotis communis	◯	-2
▪ Bifurkation A. carotis communis	◯	-1
▪ V. jugularis interna	◯	-1
▪ M. sternocleidomastoideus	◯	-2
▪ **Bonuspunkt:** M. omohyoideus	◯	**+1**
**Abzug gesamt:**		**/ 8**
**Bonuspunkte:**		**/ 1**

4) Schnitt

**Regio submandibularis**	falsch	Abzüge
• Schallkopfhaltung horizontal	◯	-2
• Darstellung:		
▪ Gl. parotis	◯	-1
▪ Gl. submandibularis	◯	-2
▪ A. facialis	◯	-1
▪ Zungenbinnenmuskulatur	◯	-1
**Abzug gesamt:**		**/ 7**

5) Schnitt

**Regio parotidea**	falsch	Abzüge
• Schallkopfhaltung horizontal	◯	-2
• Schallkopfhaltung vertikal	◯	-2
• Darstellung:		
▪ Gl. parotis (KW & präauriculär)	◯	-2
▪ Mandibula (distale Auslöschung)	◯	-1
▪ M. masseter (inkl. Testung)	◯	-2
▪ **Bonuspunkt:** Übers. GP/MM/UK	◯	**+1**
**Abzug gesamt:**		**/ 9**
**Bonuspunkt:**		**/ 1**

6) Schnitt

**Hintere Halsabschnitte**	falsch	Abzüge
• Schallkopfhaltung vertikal	◯	-2
• Darstellung:		
▪ ACC	◯	-2
▪ V. jugularis interna	◯	-1
▪ M. sternocleidomastoideus	◯	-2
▪ **Bonuspunkt**: Wirbelkörper	◯	**+1**
**Abzug gesamt:**		**/ 7**
**Bonuspunkte:**		**/ 1**

Reihenfolge Gesamtuntersuchung ◯ **-3** **Aufforderung zu Untersuchung nötig** ◯ **-2**

Abzug gesamt: _____ Erreichte Punkte: _____ / 61 Bonuspunkte _____ /11
